# Guess who? On the importance of using appropriate name: case study of *Marphysasanguinea* (Montagu, 1813)

**DOI:** 10.3897/zookeys.859.34117

**Published:** 2019-07-02

**Authors:** Nicolas Lavesque, Guillemine Daffe, Jacques Grall, Joana Zanol, Benoit Gouillieux, Pat Hutchings

**Affiliations:** 1 Univ. Bordeaux, EPOC, UMR 5805, Station Marine d’Arcachon, 33120 Arcachon, France Université de Bordeaux Arcachon France; 2 CNRS, EPOC, UMR 5805, Station Marine d’Arcachon, 33120 Arcachon, France Station Marine d’Arcachon Arcachon France; 3 CNRS, Université de Bordeaux, Observatoire Aquitain des Sciences de l’Univers, UMS 2567 POREA, 33615 Pessac, France Université de Bordeaux Pessac France; 4 Université de Brest, CNRS, UMS 3113, Observatoire, Séries Faune-Flore, OSU-IUEM, 29280 Plouzané, France Université de Brest Plouzané France; 5 Laboratório de Biodiversidade de Annelida, Departamento de Invertebrados, Museu Nacional, Universidade Federal do Rio de Janeiro, Rio de Janeiro, Brazil Universidade Federal do Rio de Janeiro Rio de Janeiro Brazil; 6 Australian Museum Research Institute, Australian Museum, Sydney, Australia Australian Museum Sydney Australia; 7 Department of Biological Sciences, Macquarie University, North Ryde 2109, Australia Macquarie University North Ryde Australia

**Keywords:** Bait worms, cosmopolitan species, misidentification, molecular, taxonomy

## Abstract

The common bait worm *Marphysasanguinea* (Montagu, 1813), originally described from the south coast of England, is the type species of the genus. This species has been widely reported from all around the world and has been considered as cosmopolitan until recently. This is partly because the original description was very brief and poorly illustrated, and also because all species superficially look similar. In order to clarify the situation, *M.sanguinea* was redescribed and a neotype was designated by Hutchings and Karageorgpoulos in 2003. Recently, specimens from Cornwall, close to the type locality, were sampled, examined morphologically, and used to obtain COI gene sequences for this species. Molecular results permitted us to confirm the identity and presence of *M.sanguinea* along the French coasts and to highlight the presence of inaccurate sequences of this species on GenBank. Use of this “false” cosmopolitan species at a worldwide scale by many biologists is also discussed in this paper.

## Introduction

Eunicidae Berthold, 1827 is a very speciose family with eleven recent genera and more than 400 valid species distributed worldwide (Read and Fauchald 2019a). The genus *Marphysa* de Quatrefages, 1866 comprises approximately 70 valid species (Read and Fauchald 2019b) and many of these have similar general morphology. *Marphysasanguinea* (Montagu, 1813), type species of the genus, has a brief and poorly illustrated original description, which could fit most species of the genus. Thus, *M.sanguinea* has been considered for decades as a cosmopolitan species (Hutchings and Kupriyanova 2017). Indeed, this species was reported from Europe (Fauvel 1923; Parapar et al. 1993; Lewis and Karageorgopoulos 2008; Hutchings et al. 2012), Grand Caribbean Region (Salazar-Vallejo and Carrera-Parra 1998), Pacific and Atlantic coasts of North America (Leidy 1855; Webster 1879; Hartman 1944; Fauchald 1970), Atlantic Coast of South America (Morgado and Tanaka 2001), Red Sea (Fauvel 1953), Africa (Day 1967; Kouadio et al. 2008; Lamptey and Armah 2008), Asia (Miura 1977), and Australia (Day 1967).

In the absence of type material, Hutchings and Karageorgopoulos (2003) decided to clarify the status of this species and described a neotype. They provided a complete description of specimens from the type locality (Cornwall, England) together with SEM plates and data about habitat and reproduction. Subsequent to this work, several species previously identified as *M.sanguinea* at a worldwide scale were carefully checked and some described as new species: *Marphysamullawa* Hutchings & Karageorgopoulos, 2003 (from Australia), *Marphysaelityeni* Lewis & Karageorgopoulos, 2008 (from South Africa), *Marphysakristiani* Zanol, da Silva & Hutchings, 2016 (from Australia), *Marphysavictori* Lavesque, Daffe, Bonifácio & Hutchings, 2017 (from France), *Marphysahongkongensa* Wang, Zhang & Qiu, 2018 (from Hong-Kong), *Marphysaaegypti* Elgetany, El-Ghobashy, Ghoneim & Struck, 2018 (from Egypt), and also a suite of species from China where most previous records recorded *M.sanguinea* as being present: *Marphysamultipectinata*, *Marphysatribranchiata* and *Marphysatripectinata* Liu, Hutchings & Sun, 2017, *Marphysabulla* Liu, Hutchings & Kupriyanova, 2018, *Marphysamaxidenticulata* Liu, Hutchings & Kupriyanova, 2018. Molina-Acevedo and Carrera-Parra (2015) also refuted the presence of *M.sanguinea* in the Grand Caribbean region. All these works confirm the absence of *M.sanguinea* outside European waters. Most of these recent studies provide molecular data for type specimens and compare them to sequences stored in GenBank (NCBI), including sequences of *M.sanguinea* from several localities, but none from the type locality.

In this study, we test the identification of *M.sanguinea* cytochrome oxidase I (COI) sequences in GenBank, comparing them with those of specimens from the type locality (Cornwall, UK). We have also carefully checked and described the studied material.

## Materials and methods

### Sampling and morphological analyses

Specimens were collected in subtidal turf slabs in Arcachon Bay, in intertidal soft rocks in Bay of Brest (France) and in rocks easily split to extract the worms in Plymouth Sound (Cornwall, UK), close to the type locality. Specimens from Brest and Cornwall were fixed and preserved in 96% ethanol. For the Arcachon specimen, several posterior parapodia were removed and fixed in 96% ethanol for molecular studies. The rest of specimen was fixed in 4% formaldehyde seawater solution, then transferred to 70% ethanol for morphological analyses. Preserved specimens were examined under a Nikon SMZ25 stereomicroscope and a Nikon Eclipse E400 microscope and photographed with a Nikon DS-Ri 2 camera. Measurements were made with the NIS-Elements Analysis software. Selected parapodia along the body were removed from one specimen from Brest (AM W.49086) and examined under the scanning electron microscope (JEOL JSM 6480LA) and imaged with a secondary detector at Macquarie University, Sydney, Australia.

Morphological terminology is based on previous studies of Paxton (2000) and Zanol et al. (2014) for general terms and pattern of subacicular hook colour, and Molina-Acevedo and Carrera-Parra (2015, 2017) for jaw morphology and for description of chaetae.

The studied material is deposited at the Australian Museum, Sydney (**AM**), National Museum of Brazil, Rio de Janeiro (**MNRJ**) and the Muséum National d’Histoire Naturelle, Paris (**MNHN**).

### Molecular data and analyses

Sub-samples for DNA analysis were removed from specimens, placed in ethanol 96% and frozen at -20 °C. Extraction of DNA was done with QIAamp DNA Micro Kit (QIAGEN) following protocol supplied by the manufacturers. Approximately 600 bp of COI (cytochrome c oxidase subunit I) gene was amplified, using primers polyLCO and polyHCO COI (Carr et al. 2011). PCR (Polymerase Chain Reaction) occurred in 50 μL mixtures containing: 10μL of 5X Colorless GoTaq Reaction Buffer (final concentration of 1X), 1.5 μL of MgCl_2_ solution (final concentration of 1.5mM), 1 μL of PCR nucleotide mix (final concentration of 0.2 mM each dNTP), 0.5 μl of each primer (final concentration of 1μM), 0.2 μl of GoTaq G2 Flexi DNA Polymerase (5U/μl), 1 μl template DNA and 33.8 μL of nuclease-free water. The temperature profile was as follows for 16S: 94 °C/600s - (94 °C/60s-59 °C/30s-72 °C/90s)*40 cycles - 72 °C/600s - 4 °C, for COI: 94 °C/600s - (94 °C/40s-44 °C/40s-72 °C/60s)*5 cycles - (94 °C/40s-51 °C/40s-72 °C/60s)*35 cycles - 72 °C/300s - 4 °C. PCR success was verified by electrophoresis in a 1 % p/v agarose gel stained with ethidium bromide. Amplified products were sent to GATC Biotech Company to complete double strain sequencing, using same set of primers as used for PCR.

Overlapping sequence (forward and reverse) fragments were merged into consensus sequences and aligned using Clustal Omega. COI sequences were translated into amino acid alignment and checked for stop codons in order to avoid pseudogenes. The minimum length coverage was around 590 bp.

Pairwise Kimura 2-parameter (K2P) genetic distance and Maximum Likelihood tree using K2P model and non-parametric bootstrap branch support (1000 replicates) was performed using MEGA version 7.0.26. Tree-based analysis was obtained with all *Marphysa* species and available (and exploitable) sequences of *M.sanguinea* in GenBank. Other genera of Eunicidae were considered as outgroup.

## Results

### Taxonomic Account

#### Family Eunicidae Berthold, 1827

##### Genus *Marphysa* Quatrefages, 1866

**Type species.***Nereissanguinea* Montagu, 1813

###### Marphysa sanguinea

Taxon classificationAnimaliaEunicidaEunicidae1

(Montagu, 1813)

Figs 1 , 2 , 3

####### Material examined.

MNHN-IA-TYPE 1856, one complete specimen, Mount Edgcumbe, Plymouth Sound, Cornwall, UK (50°20'59"N, 4°09'52"W), intertidal in soft rocks, 04 November 2017. MNRJP002048, one complete specimen, Mount Edgcumbe, Plymouth Sound, Cornwall (UK) (50°20'59"N, 4°09'52"W), intertidal in soft rocks, 04 November 2017. AM W.51410, one complete specimen, Mount Edgcumbe, Plymouth Sound, Cornwall (UK) (50°20'59"N, 4°09'52"W), intertidal in soft rocks, 04 November 2017. MNHN-IA-TYPE 1857, one complete specimen, Pyla, Arcachon Bay, France (44°33'57"N, 1°14'16"W), subtidal in turf slab (8m depth), 29 October 2017. AM W. 49085, one complete specimen, Logonna-Daoulas, Bay of Brest, France (48°19'37"N, 4°19'27"W), intertidal in soft rocks, 18 October 2016. AM W.49086, Logonna-Daoulas, Bay of Brest, France (48°19'37"N, 4°19'27"W), intertidal in soft rocks, 18 October 2016, several parapodia mounted for SEM. AM W. 27392, one complete specimen, Devon, Plymouth, Mount Edgcumbe (50°21'10"N, 4°09'30"W), intertidal from burrows in rock crevices, 25 October 1999.

####### Description.

**Figure 1.**
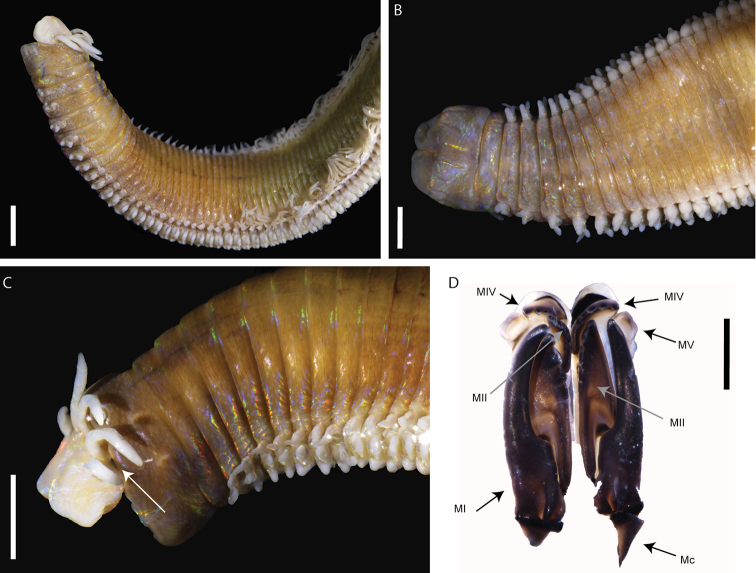
*Marphysasanguinea*: **A** anterior part, dorsolateral view (MNHN-IA-TYPE 1856) **B** anterior part, ventral view (MNHN-IA-TYPE 1856) **C** anterior part, lateral view (MNRJP002048) **D** Maxillae, dorsal view (MNHN-IA-TYPE 1856). Key: white arrow showing eye; MI to MV, maxillae I to V, Mc, maxillary carriers. Scale bars: 2 mm (**A–C**), 1mm (**D**).

Body relatively long, with complete individuals ranging from 48.1 (ca. 138 chaetigers) to 163.1 mm (ca. 270 chaetigers) in length and from 3.7 to 6.6 mm in width (chaetiger 10 with parapodia), with same width throughout, slightly tapering at anterior end and abruptly tapering at posterior end. Body cylindrical on anterior chaetigers, becoming dorsoventrally flattened. Prostomium slightly shorter than anterior ring of peristomium, as wide as peristomium, bilobed with buccal lips separated by deep ventral and dorsal notch with each lobe rounded (Fig. 1B, C). Anterior ring of peristomium longer than posterior ring (2 to 3 times) (Fig. 1B, C). Eyes present, positioned posteriorly between palps and lateral antennae (Fig. 1C). Prostomial appendages slightly wrinkled, arranged in arc on the posterior margin of the prostomium; median antenna longer than lateral antennae reaching first chaetiger (Fig. 1A), palps shortest appendages (Fig. 1A, C). MI more than three times as long as carrier and five times longer than closing system. MIII located ventroanterior to MII. Attachment lamella of MIII long and thin, placed at the middle of the plate. Left MIV with attachment lamella semicircular, thin, situated along anterior edge. Right MIV with attachment lamella semicircular, larger than left one, situated along anterior edge. Maxillary formula: I=1+1, II=3‒4+5, III=6-7+0, IV=4+5‒6, V=1+1 (Fig. 1D).

**Figure 2.**
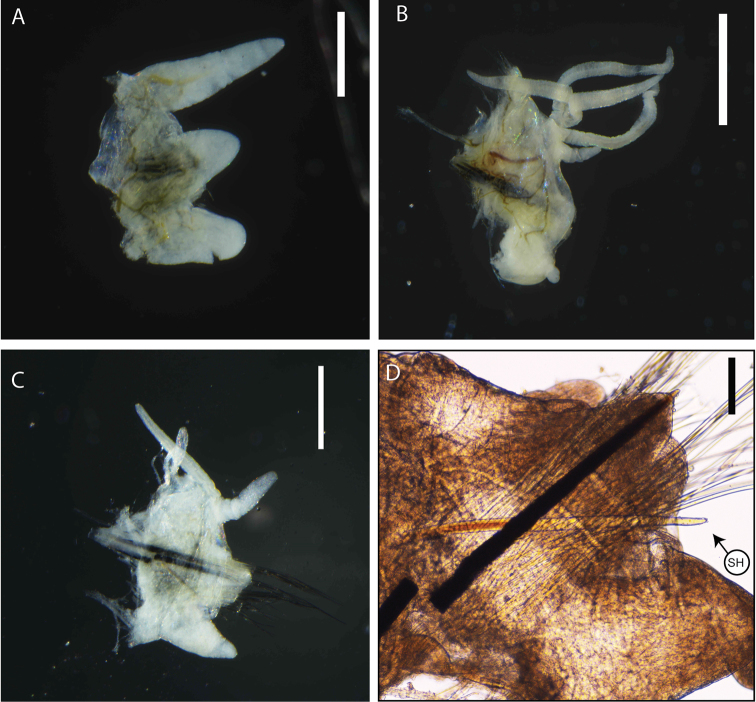
*Marphysasanguinea* (MNHN-IA-TYPE 1856): **A** parapodium from anterior chaetiger **B** parapodium from mid-body **C** parapodia from posterior chaetiger **D** parapodium from posterior chaetiger. Abbreviation: SH, Subacicular hook. Scale bars: 1 mm (**B**), 500µm (**A, C**), 100µm (**D**).

First few parapodia smaller than subsequent ones but all similar in structure. Notopodial cirri elongate and triangular (Figs 1C, 2A), digitiform in last chaetigers (Fig. 2C); longer than chaetal lobe. Ventral cirri from chaetiger 1 to 4–5 conical to tapering, with round wide tips, shorter than notopodial cirri (Fig. 2A); basally inflated from chaetiger 5–6, inflated base of round shape with round tip (Figs 1B, 2B); last chaetigers with triangular cirri (Fig. 2C). Pre-chaetal lobe inconspicuous; post-chaetal lobe from first chaetigers triangular swollen (Fig. 2A), longer than chaetal lobe, becoming inconspicuous from ca. chaetigers 15–20 (Figs 2B, C). Branchiae pectinate, from chaetiger 21 (from chaetiger 13 for small specimens) (Figs 1A, 2B), extending posteriorly by last 5–15 chaetigers; number of branchial filaments increasing from one in first chaetigers to maximum four in mid-body (Fig. 2B), posterior chaetigers with two filaments; filaments slightly annulated.

**Figure 3.**
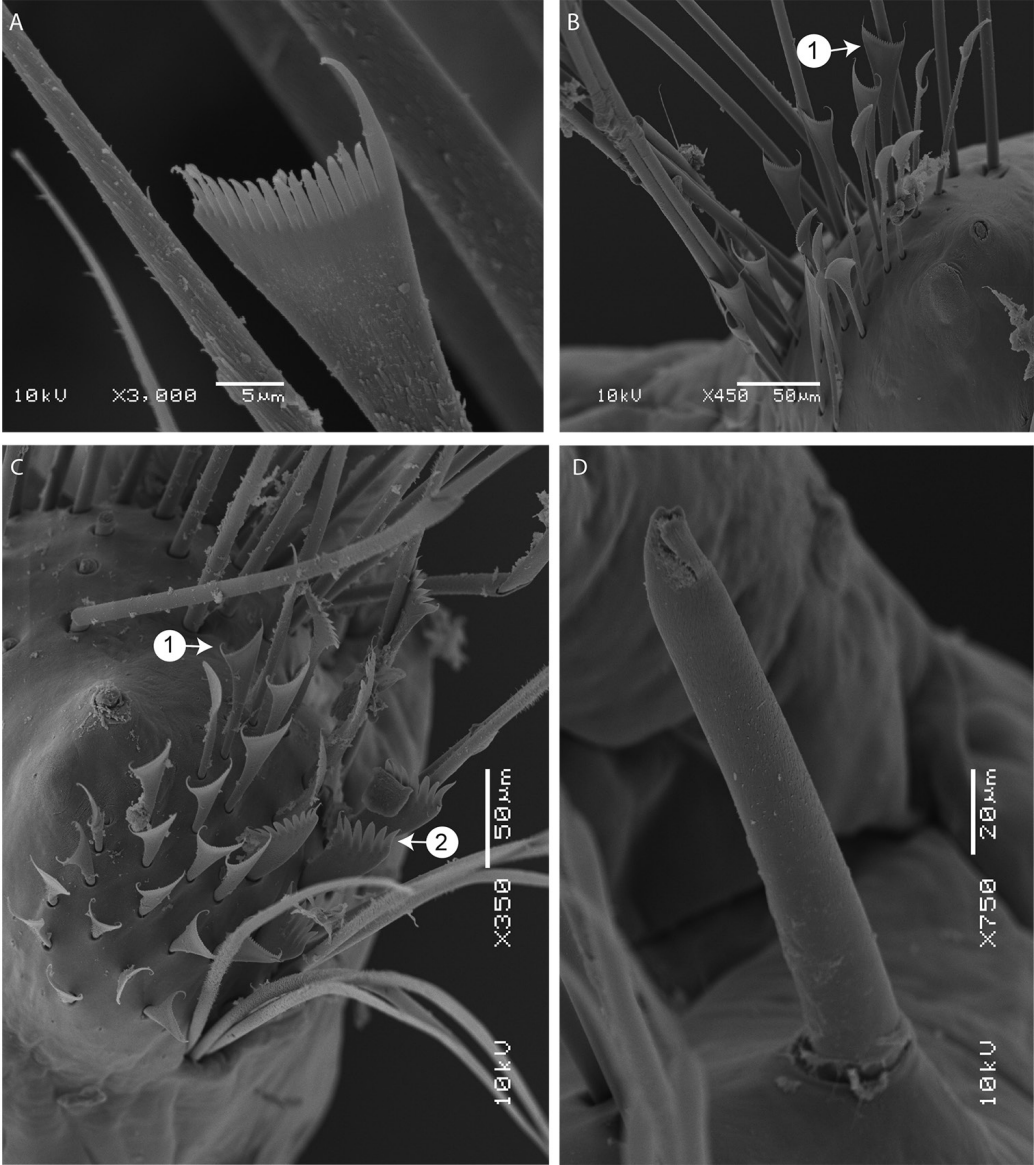
SEM images of *Marphysasanguinea*: **A** isodont, symmetrical pectinate chaetae from anterior chaetiger (AM W.49086, 3^rd^ chaetiger) **B** isodont, symmetrical pectinate chaetae from mid-body chaetiger (AM W.49086, chaetiger 108) **C** the two types of pectinate chaetae (AM W.49086, far posterior chaetiger) **D** subacicular bifid hook (AM W.49086, chaetiger 142). Numbers in white circles indicate the type of pectinate chaetae.

Chaetae arranged in two bundles: supra-acicular and sub-acicular, separated by a row of aciculae. Aciculae dark, tapering, very protruding, 1–4 per parapodium in anterior chaetigers and 2–3 in mid and posterior chaetigers. Single subacicular bifid hook present from chaetiger 21–25 to nearly end of body, dark on base to middle and translucent at the distal end (Figs 2D, 3D). Supra-acicular bundle with limbate and pectinate chaetae; sub-acicular with compound spiniger chaetae. Between 10 to 20 limbate chaetae, chaetae of different lengths with hirsute blades, similar to each other. Pectinate chaetae present from chaetiger 2–3 (with up to 28 pectinate chaetae within a single parapodia), restricted to supra-acicular fascicle. Pectinate chaetae of two types. In anterior parapodia, isodonts narrow (n < 10) with long internal teeth (with ca. 14–15 tapering teeth) and two long outer winged teeth (nearly 2–3 times longer than inner teeth) (type 1) (Fig. 3A). Median and posterior parapodia with two types of pectinate chaetae (Fig. 2C): thin, isodonts narrow, with ca. 25 short teeth (type 1) (Fig. 3B, C); anodonts wide pectinate chaetae with long and thick teeth (n = 6–14) (type 2) (Fig. 3C); Type 2 less numerous (3–7) than type 1 (16–22). Compound spinigers with hirsute shafts and “socket-like” articulations (Fig. 2A), present along whole body, with more than 30 spinigers within a parapodia. Compound falcigers absent.

Pygidium with only one pair of relatively short pygidial cirri on ventral margin (approximately as long as last five chaetigers), anus slightly crenulated.

####### Remarks.

Specimens both from British and French coasts agree with the description of the neotype and with voucher AM W.27392 which was also compared in the neotype description by Hutchings and Karageorgopoulos (2003). Most morphological characteristics are within the variation range of those observed by Hutchings and Karageorgopoulos (2003). However, few differences can be noticed: (1) larger number of pectinate chaetae (up to 28, instead of 10–14) beginning from chaetiger 2–3 (instead of chaetiger 1–2), (2) presence of coarsely denticulate chaetae with less teeth (6–14 teeth instead of ca. 14). These variations are typical within a species in the *Marphysa* genus.

####### Molecular data.

COI gene was successfully sequenced and published at NCBI GenBank for the tree specimens sampled in Cornwall near the locality type (Table 1). COI was also successfully sequenced for specimens sampled in Brest and in Arcachon (Table 1).

**Table 1.** List of terminal taxa used in molecular analysis, GenBank accession numbers, status of the species, locality of analysed specimen, and voucher specimen catalogue numbers.

Species	GenBank accession number	Status	Locality	Voucher specimen
Eunicecf.violaceomaculata Ehlers, 1887	GQ497542	valid	Carrie Bow Cay, Belize	
*Palolaviridis* Gray in Stair, 1847	GQ497556	valid	Kosrae, Micronesia	
*Leodicerubra* (Grube, 1856)	GQ497528	valid	Ceará, Brazil	
*M.aegypti* Elgetany, El-Ghobashy, Ghoneim & Struck, 2018	MF196968	valid	Suez Canal, Egypt	
*M.bifurcata* Kott, 1951	KX172177	valid	Lizard Island, Australia	
*M.brevitentaculata* Treadwell, 1921	GQ497548	valid	Quintana Roo, Mexico	
*M.californica* Moore, 1909	GQ497552	valid	California, USA	
*M.disjuncta* Hartman, 1961	GQ497549	valid	California, USA	
*M.fauchaldi* Glasby & Hutchings, 2010	KX172165	valid	North Australia	
*M.kristiani* Zanol et al., 2016	KX172141	valid	Cowan Creek, Australia	
*M.mossambica* (Peters, 1854)	KX172164	valid	Australia	
*M.mullawa* Hutchings & Karageorgopoulos, 2003	KX172166	valid	Careel Bay, Australia	
*M.pseudosessiloa* Zanol, da Silva & Hutchings, 2017	KY605405	valid	Careel Bay, Australia	
*M.victori* Lavesque, Daffe, Bonifácio & Hutchings, 2017	MG384997	valid	Arcachon, France	
*M.viridis* Treadwell, 1917	GQ497553	valid	Ceará, Brazil	
*M.sanguinea* (Montagu, 1813)	GQ497547	valid	Callot Island, France	
	MK541904	valid	Cornwall, UK	AM W.51410
	MK950851	valid	Cornwall, UK	MNHN-IA-TYPE 1856
	MK950852	valid	Cornwall, UK	MNRJP002048
	MK950853	valid	Arcachon, France	MNHN-IA-TYPE 1857
	MK967470	valid	Brest, France	AM W. 49085
	MH826265	invalid	USA	
	KP255196	invalid	USA	
	KR916873	invalid	Portugal	
	AY040708	invalid	?	
	KY129890	invalid	East China Sea	
	KY129891	invalid	East China Sea	
	KF733802	invalid	Yellow Sea, China	
	EU352317	invalid	China?	
	EU352316	invalid	China?	

**Figure 4.**
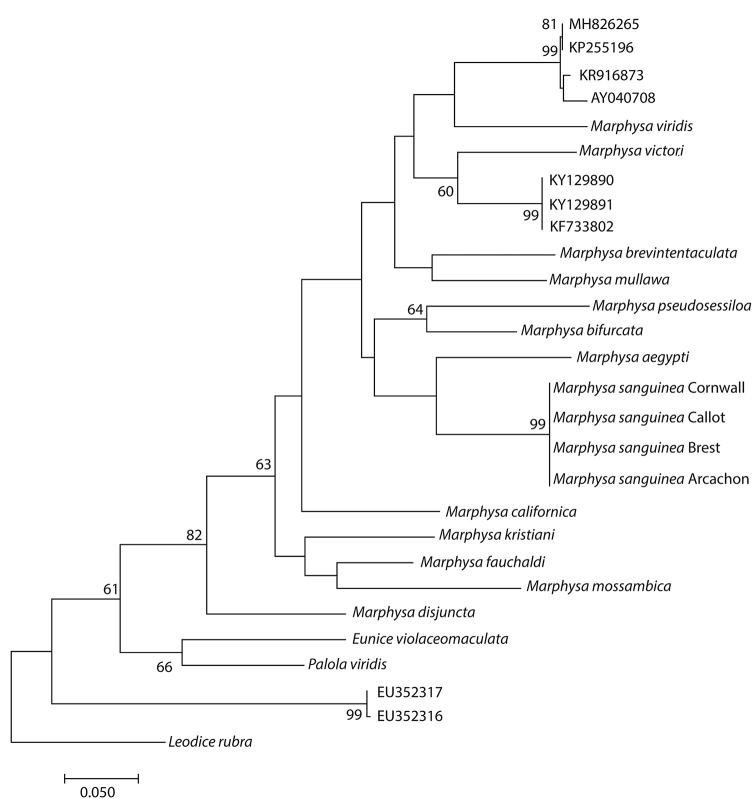
Maximum Likelihood tree of valid species of *Marphysa* and different *Marphysasanguinea* available in GenBank, based on cytochrome oxidase I (COI) sequences and Kimura-2-parameters model. Bootstrap values on nodes if >50. Sequence accession numbers refer to Table 1.

First of all, molecular analysis distinguished *M.sanguinea* from other species with sequences available in GenBank (Fig. 4). Analysis permitted the grouping of specimens of *M.sanguinea* from Cornwall together with specimens from French Atlantic coast (Arcachon, Brest) but also from southern English Channel, Callot Island (Zanol et al. 2014) (Fig. 4). Intraspecific pairwise genetic distances for COI were zero among these specimens. This tree clearly emphasised the presence of different species among this *sanguinea* complex. Especially, some specimens registered as *M.sanguinea* did not belong even to the *Marphysa* genus (EU352317 and EU352316).

Finally, a comparison of sequences of COI of a specimen from the type locality (AM W.51410) with specimen used to sequence the complete mitochondrial genome of *M.sanguinea* (accession number: KF733802, specimen from China) (Li et al. 2016) was performed. Unsurprisingly, these sequences were very different; the interspecific pairwise genetic distance was 18.5%.

## Discussion

This study provides a molecular baseline for future taxonomic works. Among the *M.sanguineasequences* in GenBank, molecular analyses only confirmed the identification of sequence GQ497547 (Zanol et al. 2014) from coarse sand near a *Zosteramarina* seagrass bed in Callot Island (English Channel, northern Bretagne, France). All other sequences are not *M.sanguinea* and K2P genetic distance between these sequences and the specimen from the type locality varied from 13.6% (with KR916873) to 35.1% (with EU352316).

This study, therefore, confirms the presence of *M.sanguinea* along the French coasts, from the English Channel to the Bay of Biscay. Except for specimens from the French part of the English Channel (Zanol et al. 2014), which were sampled in coarse sand, all the confirmed records of *M.sanguinea* indicate that they are often associated with hard substrates. Specimens from the type locality (this study, Hutchings and Karageorgopoulos 2003) lived intertidally, in deep burrows in crevices in rocks at low watermark. In Arcachon Bay, they were found subtidally, inside turf slabs. Finally, in the Bay of Brest, specimens were also sampled from intertidal soft rocks. Except for specimens from Callot, all studied specimens were sampled in hard substrates. Actually, *Marphysa* species are known to occur in a range of specific habitats: muddy seagrass beds (e.g., *M.mullawa* (Hutchings and Karageorgopolous 2003, Zanol et al. 2016)), muddy flats (e.g., *M.kristiani* (Zanol et al. 2016)), sandy shores (*M.hongkongensa* (Wang et al. 2018), aquaculture fish ponds (e.g., *M.fauchaldi* (Glasby and Hutchings 2010)), oyster reefs (e.g., *M.victori* (Lavesque et al. 2017)).

Among the GenBank sequences that have been misidentified as *M.sanguinea*, the most astonishing is the sequence that is part of the complete mitochondrial genome of a species from the coast of the Yellow Sea (China) (GenBank accession number: KF733802) (Li et al. 2016). This species forms a monophyletic clade with other sequences from East China, suggesting that either a new species is present in this area or specimens belong to a described species for which there is no sequence identified as such in GenBank. Moreover, we also found an alarming result with the presence in GenBank of sequences registered as *M.sanguinea* which did not even belong to the genus *Marphysa* (EU352317 and EU352316). This finding confirms the necessity of cautiously using these sequences, because these sequences come from specimens that clearly do not belong to *M.sanguinea*, and inevitably continues the confusion regarding the identity of this species. Furthermore, no vouchers were deposited in a museum that would allow for examination and comparison with other close species, or allow corroboration that it might be a new species for science. We strongly recommend verification of sequence publication in an international journal, whether a polychaete taxonomist has been associated with the study and whether a voucher specimen has been deposited in an official collection, before using the sequences.

As well as being (wrongly) considered as a cosmopolitan species for decades (Hutchings and Kupriyanova 2017), specimens identified as *M.sanguinea* are also widely used as a biological model by many scientists, but never with specimens originating from the type locality or its vicinity. Thus, many studies use specimen under the name *M.sanguinea* as a model in biochemistry, such as studies on galactosylceramides (Noda et al. 1992; Noda et al. 1994, specimens from fishing shops, Japan), erythrocruorin (Chew et al. 1965, specimens from Swan River, Australia; Weber et al. 1978, specimens from Pivers Island, North Carolina), lectins (Ozeki et al. 1997, specimens from fishing shops, Japan), phenols (Whitfield et al. 1999, specimens from Sydney, Australia), or acetylcholine (Horiuchi et al. 2003, specimens from commercial sources, Japan). Biology and physiology from so-called *M.sanguinea* specimens are also largely studied by scientists worldwide. From the literature, we identified works on development regarding sex gonad (Yu et al. 2005, specimens from Shandong Province, China), reproduction cycle (Yu et al. 2005; Ouassas et al. 2015, specimens from Saharan area, Morocco), metabolism and excretion (Yang et al. 2015, specimens from Dalian, China). Several papers also study rearing of so-called *M.sanguinea* with effects of density on growth (Parandavar et al. 2015, specimens from South Korea) or appropriate feeding for early juvenile stages (Kim et al. 2017, specimens from South Korea). Besides Li et al. (2016), several papers focus on genetic elements of this species, such as purification, characterisation and cDNA cloning of opine dehydrogenases (Endo et al. 2007, specimens from fishing shops, Japan) or genetic diversity from different geographical populations (Zhao et al. 2016, specimens from China). Finally, a recent study deals with microplastics and the formation of plastic fragments by *M.sanguinea* inhabiting marine polystyrene debris (Jang et al. 2018, specimens from Geoje Island, South Korea). While one could consider these as anecdotal, their conclusions are likely completely wrong when it comes to the species they refer to Even closely similar morphological species might have very different life-history traits (Cole et al. 2018), internal biology and of course, DNA. Such misidentifications could also lead to management and economic problems since *Marphysa* spp. are widely harvested as bait worldwide (Cole et al. 2018). In conclusion, we highly encourage marine biologists and ecologists to collaborate with confirmed taxonomists when assigning species names to marine invertebrate specimen.

## Supplementary Material

XML Treatment for Marphysa sanguinea

## References

[B1] Berthold AA (1827) Latreille’s Natürliche Familien des Thierreichs. Aus dem Französischen, mit Anmerkungen und Zusätzen. Verlage Landes-Industrie-Comptoirs, Weimar, 606 pp 10.5962/bhl.title.11652

[B2] Carr CM Hardy SM Brown TM Macdonald T Hebert PDN (2011) A tri-oceanic perspective: DNA barcoding reveals geographic structure and cryptic diversity in Canadian polychaetes. PLoS ONE 6: e22232. 10.1371/journal.pone.0022232 PMC3136506 21829451

[B3] Chew MY Scutt PB Oliver IT Lugg JWH (1965) Erythrocruorin of *Marphysasanguinea*: Isolation and some physical, physiochemical and other properties. Biochemestry Journal 94: 378–383. 10.1042/bj0940378

[B4] Cole VJ Chick RC Hutchings PA (2018) A review of global fisheries for polychaete worms as a resource for recreational fishers: diversity, sustainability and research needs. Reviews in Fish Biology and Fisheries 28: 543–565. 10.1007/s11160-018-9523-4

[B5] Day JH (1967) A Monograph on the Polychaeta of Southern Africa. Part I. Errantia. British Museum (Natural History), London, 458 pp 10.5962/bhl.title.8596

[B6] Elgetany AH El-Ghobashy AE Ghoneim AM Struck TH (2018) Description of a new species of the genus *Marphysa* (Eunicidae), *Marphysaaegypti* sp. n., based on molecular and morphological evidence. Invertebrate Zoology 15(1): 71–84.

[B7] Endo N Kan-No N Nagahisa E (2007) Purification, characterization, and cDNA cloning of opine ehydrogenases from the polychaete rockworm *Marphysasanguinea*. Comparative Biochemistry and Physiology Part B: Biochemistry and Molecular Biology 147: 293–307. 10.1016/j.cbpb.2007.01.018 17350870

[B8] Fauchald K (1970) Polychaetous annelids of the families Eunicidae, Lumbrineridae, Iphitimidae, Arabellidae, Lysaretidae and Dorvilleidae from Western Mexico. Allan Hancock Monographs in Marine Biology 5: 1–335. 10.5479/si.00810282.221

[B9] Fauvel P (1923) Polychètes errantes. Faune de France. Librairie de la Faculté des Sciences, Paris, 488 pp.

[B10] Fauvel P (1953) The fauna of India including Pakistan, Ceylon, Burma and Malaya: Annelida, Polychaeta. The Indian Press, Ltd, Allahabad, 507 pp.

[B11] Glasby CJ Hutchings PA (2010) A new species of *Marphysa* Quatrefages, 1865 (Polychaeta: Eunicida: Eunicidae) from northern Australia and a review of similar taxa from the Indo-West Pacific, including the genus *Nauphanta* Kinberg, 1865. Zootaxa 2352: 29–45. 10.11646/zootaxa.2352.1.2

[B12] Hartman O (1944) Polychaetous Annelids, 5. Eunicea. Allan Hancock Pacific Expeditions 10: 1–238.

[B13] Horiuchi Y Kimura R Kato N Fujii T Seki M Endo T Kato T Kawashima K (2003) Evolutional study on acetylcholine expression. Life Sciences 72: 1745–1756. 10.1016/S0024-3205(02)02478-5 12559395

[B14] Hutchings PA Karageorgopoulos P (2003) Designation of a neotype of *Marphysasanguinea* (Montagu, 1813) and a description of a new species of *Marphysa* from Eastern Australia. Hydrobiologia 496: 87–94. 10.1023/A:1026124310552

[B15] Hutchings PA Glasby CJ Wijnhoven S (2012) Note on additional diagnostic characters of *Marphysasanguinea* (Montagu, 1813) (Annelida: Eunicida: Eunicidae), a recently introduced species in Netherlands. Aquatic Invasions 7: 277–282. 10.3391/ai.2012.7.2.014

[B16] Hutchings PA Kupriyanova E (2017) Cosmopolitan polychaetes – fact or fiction? Personal and historical perspectives. Invertebrate Systematics. 10.1071/IS17035

[B17] Jang M Shima WJ Myung GH Song YK Hong SH (2018) Formation of microplastics by polychaetes (*Marphysasanguinea*) inhabiting expanded polystyrene marine debris. Marine Pollution Bulletin 131: 365–369. 10.1016/j.marpolbul.2018.04.017 29886959

[B18] Kim KH Kim BK Kim SK Phoo WW Maran BAV Kim CH (2017) Appropriate feeding for early juvenile stages of eunicid polychaete *Marphysasanguinea*. Fisheries and Aquatic Sciences 18: 57–63. 10.1186/s41240-017-0064-x

[B19] Kouadio KN Diomande D Ouattara A Kone YJM Gourene G (2008) Taxonomic diversity and structure of benthic macroinvertebrates in Aby Lagoon (Ivory Coast, West Africa). Pakistan Journal of Biological Sciences 20: 19. 10.3923/pjbs.2008.2224.2230 19137831

[B20] Lamptey E Armah AK (2008) Factors affecting macrobenthic fauna in a tropical hypersaline coastal lagoon in Ghana, West Africa; 31: 1006–1019. 10.1007/s12237-008-9079-y

[B21] Lavesque N Daffe G Bonifácio P Hutchings PA (2017) A new species of the *Marphysasanguinea* complex from French waters (Bay of Biscay, NE Atlantic) (Annelida, Eunicidae). ZooKeys 716: 1–17. 10.3897/zookeys.716.14070 PMC5740441 29290704

[B22] Leidy J (1855) Contributions towards a knowledge of the marine Invertebrate fauna of the coasts of Rhode Island and New Jersey. Journal of the Academy of Natural Sciences of Philadelphia, New Series 3: 135–152.

[B23] Lewis C Karageorgopoulos P (2008) A new species of *Marphysa* (Eunicidae) from the Western Cape of South Africa. Journal of the Marine Biological Association of the United Kingdom 88: 277–287. 10.1017/S002531540800009X

[B24] Li S Chen Y Zhang M Bao X Li Y Teng W Liu Z Fu C Wang Q Liu W (2016) Complete mitochondrial genome of the marine polychaete, *Marphysasanguinea* (Polychaeta, Eunicida). Mitochondrial DNA Part A 27: 42–43. 10.3109/19401736.2013.869686 24438272

[B25] Liu Y Hutchings PA Sun S (2017) Three new species of *Marphysa* Quatrefages, 1865 (Polychaeta: Eunicida: Eunicidae) from the south coast of China and redescription of *Marphysasinensis* Monro, 1934. Zootaxa 4263(2): 228–250. 10.11646/zootaxa.4377.2.3 28609867

[B26] Liu Y Hutchings P Kupriyanova E (2018) Two new species of *Marphysa* Quatrefages, 1865 (Polychaeta: Eunicida: Eunicidae) from northern coast of China and redescription for *Marphysaorientalis* Treadwell, 1936. Zootaxa 4377(2): 191–215. 10.11646/zootaxa.4377.2.3 29690064

[B27] Miura T (1977) Eunicid polychaetous annelids from Japan part II. Mer (Tokyo) 15: 11–31.

[B28] Molina-Acevedo IC Carrera-Parra LF (2015) Reinstatement of three Grand Caribbean species of the *Marphysasanguinea* complex (Polychaeta: Eunicidae). Zootaxa 3925(1): 37–55. 10.11646/zootaxa.3925.1.3 25781729

[B29] Molina-Acevedo IC Carrera-Parra LF (2017) Revision of *Marphysa* de Quatrefages, 1865 and some species of *Nicidion* Kinberg, 1865 with the erection of a new genus (Polychaeta: Eunicidae) from the Grand Caribbean. Zootaxa 4241: 1–62. 10.11646/zootaxa.4241.1.1 28603244

[B30] Morgado EH Tanaka MO (2001) The macrofauna associated with the bryozoan *Schizoporellaerrata* (Walters) in southeastern Brazil. Scienta Marina 65: 173–181. 10.3989/scimar.2001.65n3173

[B31] Montagu G (1813) Descriptions of several new or rare animals, principally marine, found on the south coast of Devonshire. Transactions of the Linnean Society of London 11: 18–21. 10.1111/j.1096-3642.1813.tb00035.x

[B32] Noda N Ryuichiro T Kazumoto M Kawasaki T (1992) Two novel Galactosylceramides from *Marphysasanguinea*. Tetrahedron Letters 33: 7527–7530. 10.1016/S0040-4039(00)60815-8

[B33] Noda N Tanaka R Tsujino K Takasaki Y Nakano M Nishi M Miyahara K (1994) Phosphocholine-Bonded Galactosylceramides Having a Tri-Unsaturated Long-Chain Base from the Clam Worm, *Marphysasanguinea*. Journal of Biochemistry 116: 435–442. 10.1093/oxfordjournals.jbchem.a124543 7822265

[B34] Ouassas M Lefrere L Ait Alla A Agnaou M Gillet P Moukrim A (2015) Reproductive cycle of *Marphysasanguinea* (Polychaeta: Eunicidae) in a Saharan wetland: Khnifiss Lagoon (South of Morocco). Journal of Materials and Environmental Science 6: 246–253.

[B35] Ozeki Y Tazawa E Matsui T (1997) d-Galactoside-Specific Lectins from the Body Wall of an Echiuroid (*Urechisunicinctus*) and Two Annelids (*Neanthesjaponica* and *Marphysasanguinea*). Comparative Biochemistry and Physiology Part B: Biochemistry and Molecular Biology 118: 1–6. 10.1016/S0305-0491(97)00014-X 9417987

[B36] Parapar J Besteiro C Urgorri V (1993) Taxonomy and Ecology of Annelida of the Iberian Peninsula – Polychaeta from the Ria-De-Ferrol. Cahiers de Biologie Marine 34: 411–432.

[B37] Parandavar H Kim KH Kim CH (2015) Effects of rearing density on growth of the polychaete rockworm *Marphysasanguinea*. Fisheries and Aquatic Sciences 18: 57–63. 10.5657/FAS.2015.0057

[B38] Quatrefages A (1866) Histoire naturelle des Annelés marins et d’eau douce. Annélides et Géphyriens. Tome second. Librairie Encyclopédique de Rôret, Paris, 337–794. 10.5962/bhl.title.122818

[B39] Read G Fauchald K (2019a) World Polychaeta database. Eunicidae Berthold, 1827. World Register of Marine Species. http://www.marinespecies.org/aphia.php?p=taxdetails&id=966 [Accessed on 2019-04-25]

[B40] Read G Fauchald K (2019b) World Polychaeta database. *Marphysa* Quatrefages, 1866. World Register of Marine Species. http://www.marinespecies.org/aphia.php?p=taxdetails&id=129281 [Accessed on 2019-02-25]

[B41] Salazar-Vallejo SI Carrera-Parra LF (1998) Eunicids (Polychaeta) from the Mexican Caribbean with keys to Great Caribbean species: *Fauchaldius*, *Lysidice*, *Marphysa*, *Nematonereis* and *Palola*. Revista de Biologia Tropical 45: 1481–1498.

[B42] Wang Z Zhang Y Qiu JW (2018) A New Species in the *Marphysasanguinea* complex (Annelida, Eunicidae) from Hong Kong. Zoological Studies 57(48): 1–13. 10.6620/ZS.2018.57-48 PMC6517775 31966288

[B43] Weber RE Bonaventura J Sullivan B Bonaventura C (1978) Oxygen equilibria and ligand-binding kinetics of erythrocruorins from two burrowing Polychaetes of different modes of life, *Marphysasanguinea* and *Diopatracuprea*. Journal of Comparative Physiology 123: 177–184. 10.1007/BF00687847

[B44] Webster HE (1879) The AnnelidaChaetopoda of the Virginian coast. Transactions of the Albany Institute 9: 202–272. 10.5962/bhl.title.11296

[B45] Whitfield FB Drew M Helidoniotis F Svoronos D (1999) Distribution of bromophenols in species of marine polychaetes and bryozoans from Eastern Australia and the role of such animals in the flavor of edible ocean fish and prawns (shrimp). Journal of Agricultural and Food Chemistry 47: 4756–4762. 10.1021/jf9904719 10552886

[B46] Yang D Chan F Zhou Y Xiu Z (2015) Diel variation in metabolism and ammonia excretion of *Marphysasanguinea* (Polychaeta: Eunicidae). Chinese Journal of Oceanology and Limnology. 10.1007/s00343-016-4340-x

[B47] Yu HZ Zhu LY Zheng JS (2005) Development of sex gonad and reproduction cycle of *Marphysasanguinea*. Journal of Fishery Sciences of China 12: 669–674.

[B48] Zhao H Wang X Yang D Zhao X Li N Zhou Y (2016) An analysis of genetic diversity in *Marphysasanguinea* from different geographic populations using ISSR polymorphisms. Biochemical Systematics and Ecology 64: 65–69. 10.1016/j.bse.2015.11.002

[B49] Zanol J Halanych KM Fauchald K (2014) Reconciling taxonomy and phylogeny in the bristleworm family Eunicidae (polychaete, Annelida). Zoologica Scripta 43: 79–100. 10.1111/zsc.12034

[B50] Zanol J da Silva T dos SC Hutchings P (2016) Integrative taxonomy of *Marphysa* (Eunicidae, Polychaeta, Annelida) species of the Sanguinea-group from Australia. Invertebrate Biology 135(4): 328–344. 10.1111/ivb.12146

[B51] Zanol J da Silva T dos SC Hutchings P (2017) One new species and two redescriptions of *Marphysa* (Eunicidae, Annelida) species of the Aenea-group from Australia. Zootaxa 4268(3): 411–426. 10.11646/zootaxa.4268.3.6 28610365

